# Exploring safety at mass gathering events through the lens of three different stakeholders

**DOI:** 10.3389/fpubh.2024.1451891

**Published:** 2025-02-07

**Authors:** Alison Hutton, Martin Robertson, Jamie Ranse

**Affiliations:** ^1^Western Sydney University, Penrith, NSW, Australia; ^2^Edinburgh Napier University, Edinburgh, United Kingdom; ^3^School of Nursing and Midwifery, Griffith Health, Griffith University, Nathan, QLD, Australia

**Keywords:** event, mass gathering, safety, police, event mangement services, medical personnel

## Abstract

**Introduction:**

The work here reviews the role of those with responsibility in managing people and their safety at Mass Gathering Events (MGE); event managers, police, and medical personnel. This paper comes at a time when there is an acute need for appropriate application of psychosocial understanding and biophysical knowledge for the safe management of the social environment of MGE, and the broader community. Safety has become increasingly significant in the vocabulary of researchers, managers, funding agencies and public bodies involved in the provision of mass gathering events.

**Method:**

Focus groups were used to gain insight into MGE safety through the lens of three different groups. Through prioritising safety at mass gathering events, organisers enhance the quality of the event, protect individual and promote the long-term success of the event. Questions were centred on their understanding of safety at events, based on their experiences in MGEs.

**Results:**

Participants in this study identified many risks to event safety, including lack of risk assessment, communication, lack of ownership of risk and poor planning. Even though these risks were similar, each participant group identified their own perspective with their own ways of managing them.

**Discussion:**

The work proposes that all event stakeholders should focus on the interests of both the audience and the broader event community, with the goal of working together to foster a safe, supportive, and trusting environment. Building trust in the context of mass gathering events brings forward an opportunity for new orientation strategies. A framework for developing personal skills and community resilience for mass gathering events is shown.

## Graphical abstract

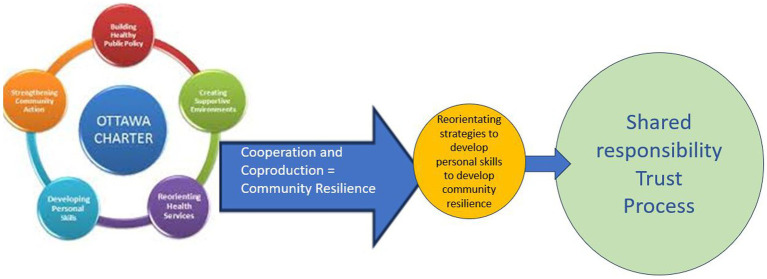

## Introduction

Key public health risks at a mass gathering event include the potential for delayed emergency response and wide-scale health effects which can occur because of access issues or environmental features, such as temperature, humidity, high decibel levels, overcrowding (1) and the passing of infection and/or viruses (2). Though the science underpinning mass gathering health and event management is developing rapidly, there is a lack of consistency around protocols and tools utilised to maintain safety at events (1, 3, 4).

A mass gathering is an event where there is: a concentration of people; at a specific location; for a specific purpose; over a defined period of time; and in sufficient numbers to potentially strain the planning and response resources of a host community, state and/or nation (5). Despite this potential for strain on the host community resources, mass gathering events are sought after and dynamic elements of social existence.

Robertson et al. (6) consider the importance of civil and social responsibility for mass outdoor events in times of socio-economic turbulence. No time is this responsibility more needed than in the turbulence that has been precipitated by COVID-19. As response to this, public events, as with all other mass activity, have been cancelled or postponed allowing for an extended period of time to pass. In so doing it is hoped contact between strangers is minimised and opportunity for the virus to travel from person to person drastically reduced. However, the influence and importance of events has not gone away.

Mass gatherings have multiple positive social impact and health benefits for attendees (7, 8). The sharing of time, the sense of goodwill and the *communitas* that organised events engender (9, 10) has been identified as socially vital. Further, outdoor music festivals and other mass gathering events are impactful on the capacity of social settings (places and people) to be resilient to change (11, 12). The work here acknowledges the importance of mass gathering events at a time of social-economic upheaval following COVID-19. It suggests that concomitantly there are opportunities for those with responsibility for their safety to co-produce event experience in ways that encourage safer experience, social resilience as well as social fulfilment for attendees and related communities. This, the researchers advance, is a response to civic need, and contributes to the civic responsibility (6, 13) that – we infer – should be a contingent for all mass gathering events at a time of social-economic turbulence, and at which socio-psychological understanding is particularly important.

The significance of mass gathering events to young people is marked (14) and the responsibilities of mass gathering event organisers and their funding agencies heightened in a time of economic and social fragility. Successful and safe event experience is “predicated on an understanding of psychosocial domain of the audience” [(3), p. 44] [see (15–17)]. It is for this reason that the research in this study reflects specifically to event safety and the importance of multiple views, interaction of people’s knowledge, to confirm social trust and capacity for real-time interaction.

Current event safety protocols are based on a risk assessment/risk management paradigm that is often part of the legislative environment of the local government area/city/state/country where the mass gathering is staged (3). Once the risk assessment has been completed and a risk management plan developed, event organisers, emergency services and other event-related safety personnel operate reactively. Planning is undertaken prior to the event to mitigate potential risks identified in the planning process, but during the event the management of incidents are reactive/responsive (that is, after the incident has occurred) rather than pre-emptive/proactive to reduce the potential for the incident to happen in the first place (3, 18). Personnel remain on call until such time as an incident occurs and only then are tasked to respond. Safety has become increasingly significant in the vocabulary of researchers, managers, funding agencies and public bodies involved in the provision of events. This work reviews the role of those with responsibility for managing people and their safety at events. These are event managers, police and medical teams’, each of whom may have different perceptions of safety whilst managing large events.

### Aim

The aim of this paper is to explore the perspective of event managers, police and medical personnel in regard to risks that can compromise event safety, and to explore what specific protocols and principles can be put in place to support the effective ongoing measurement and management of safety at a MGE.

## Methods

This study adopted a qualitative descriptive methodology (QD). This specific methodology facilities the study of the phenomena of interest without prior agenda or knowledge. Qualitative descriptive methodology seeks to characterise the viewpoints of participants in a way most people observing or witnessing the same event would provide a similar description (19). The sample included experienced event personnel such as: event managers, police, and medical personnel working in the Australian setting. A total of fifteen participants took part in the research; seven police, five medical personnel and three event managers. All participants had experience in the mass gathering event industry. The police participants were part of the special events units in a major capital city. The event managers oversaw operations at a large facility in a capital city in Australia. Lastly, the medical personnel specialised in delivering medical services specifically for mass gathering events.

### Data collection

Data collection was obtained via three focus groups. Focus groups were used to engage three groups of experienced event personnel: event managers, police, and medical personnel working in the Australian setting. A total of fifteen participants took part in the research; seven police, five medical personnel and three event managers took part in this research. Focus groups took place in the workplace of participants. A set series of questions was used to guide the focus group, and took no longer than an hour each to complete. The first author (AH) and a colleague undertook all focus groups. Focus groups have the advantage of being flexible, allowing the researcher to obtain rich data and allowing synergy of thought that may not have emerged from interviews on an individual basis (20). Focus group took place in the workplace of participants and took no longer than an hour each to complete. The aim of using focus groups was to gain insight into event safety through the lens of three different groups that work in this area. Questions were centred on their understanding of safety at events, based on their experiences in the event world and the related situational contexts (21). Key participants from each organisation were identified and approached to explore the practises they currently use to manage safety at a MGEs in each meeting, the data was condensed, and themes drawn from keyword analysis, utilising a process of content analysis and coding (22). Participants were asked to work retrospectively from an outcome (positive or negative), step by step, to the first aspect of the event that alerted them that they needed to take notice/action (and then progress to the steps in-between).

### Data analysis

Thematic analysis was used to analyse the data. An inductive, semantic approach to thematic analysis was selected as this method supported the exploratory nature of the study and ensured that the themes identified were strongly linked to the collected data (23). The analysis involved five phases: (1) familiarising oneself with the data; (2) generating initial codes as a means of indexing and categorising the transcripted text to establish the framework of thematic ideas. Typical coding labels were words and terms such as risk, safety, protocols, principles, planning and re-evaluating (3) searching for themes from codes and categories; (4) reviewing themes for accuracy and consistency; and (5) defining and naming final themes. This then allowed patterns to appear, sub-themes to be grouped and, finally, key themes to emerge (23). Using this approach, the two main organising themes that arose from the collected data were, (1)‘risks that can compromise safety’ and (2) specific protocols and principles to support effective management. To increase trustworthiness, the data and resulting analysis were individually undertaken by each member of the research team and then member-checked, where agreement on the final themes was achieved. Ethics approval for the study was obtained from the Social Behavioural Research Ethics Committee at Flinders University.

## Findings

Findings were separated into two parts. The first being identified risks that can compromise safety at the event, such as lack of risk assessment, communication, and planning. Secondly, specific protocols and principles that can be put in place to support the effective ongoing measurement and management of safety such as qualifications, mentoring, and professionalism.

### Compromising safety at an event

Lack of risk assessment, communication and planning were seen as the dominant forces that contributed to an unsafe event. In addition, *“poor planning, poor structure in place, and poor resourcing”* (Police, 6).

As we as the above-mentioned risks, a lack of ownership of risks was cited as a safety concern. For example, intoxication of attendees at an event.

*“The ownership of risks needs to be understood better, and a person cannot, or an event cannot automatically transfer risk to another agency unless there is a formal agreement in regards to it.” (Police, 1).*

*“Responsibilities and requirements need to be far more clearly identified with the event manager having to now step up and clearly sort of take control of their event more than what they used to.” (Police, 2).*

As well as individual responsibility there was a concern by participants that there was no formal oversight of events.

*“There is no central registry and neither, is there any agency or body which has an oversight of events in the* state.” (Police, 4).

In addition, a lack of formal mentoring was seen as a risk to event safety;

The bit that’s missing from me is there’s no mentoring because it’s an industry that’s quite competitive.” (Medical, 3).

The professional relationship has been built because there’s continuity, educating all of us in regards to roles and responsibilities to the extent where we are now sitting and briefing security….” (Police, 2).

### Protocols and principals

Participants discussed specific protocols to support the management of safety at events, these being qualifications, mentoring, professionalism, and working as a team;

*“We do meet with an emergency services team, so that’s the police, ambulance, Safe Work, local councils, PIRSA, animal health, those sorts of groups. We meet three times a year to talk about the show.”* (Event Manager, 3).

*“When you look at systems development in colour coding, it seems to work internationally quite well.”* (Medical, 2).

*(If monster truck catches fire) “The protocol would be that we have the fire extinguishers close at hand. Everyone is told ‘if there’s a fire and you are trained we are more than happy for you to attempt to control it.”* (Event Manager, 1)

*“Hazard management, incident reporting, those sorts of things, everything at the end of the day comes back to if* there is an incident everyone know what to do because we are so well drilled now.” (Event Manager, 2).

As well as team work prevention action was seen as fundamental to effective event safety;

“[we are] *becoming more aware* [that] *preventative actions are better than response actions and I think that really is the key, hence that’s what planning is, trying to plan in a preventative way so the event is appropriate, safe and enjoyable and you do not get all the other issues*.” (Police, 5).

Personal relationships, trust and understanding is an important component of event safety.

*“My philosophy here in running my unit is to ensure that my personnel know their jobs and know the product that they need to produce.”* (Police, 2).

*“If you have got that relationship you know you can safely delegate things to your suppliers because you know what their capabilities are versus coming in, not knowing anyone, not knowing their capabilities but knowing that you are ultimately responsible for the outcome.”* (Medical, 1).

Lastly, debriefing and continuous quality improvement was seen an integral to developing an event safety culture.

*“We encourage all event organisers in particular to debrief, and a multi-agency stakeholder debrief at the end of it, but that’s only an encouragement. We have no control as to whether they run them or not and unfortunately a vast majority do not. However, whatever we identify we will certainly bring up as a point of issue at the next event..//.., that’s where we’ll actually address it and try to have it identified and actioned accordingly.”* (Police, 3).

*At the end of each event we provide a basic report on how many patients we saw, what their presentation types were, obviously de-identified data that can fed back and be used for future planning.”* (Medical, 2).

### A way forward: planning and re-evaluating

All three groups identified possible risks to event planning, however they were able to pinpoint strategies to move forward in the event safety space.

All three groups agreed that planning, reflection and reviewing procedures is an ongoing part of event safety. The first part of planning is to,

*“what planning is, trying to plan in a preventative way so the event is appropriate, safe and enjoyable and you do not get all the other issues.”* (Police, 4).

*“…gain as much information as we can so we can make a determination in regards to what is the classification for that event, who’s going to have carriage of that event.”* (Police, 1).

Once an initial plan is made participants stated it is important to be flexible in reviewing and updating plans to ensure a safe event.

*“We’d done the initial planning, then come back and reviewed the planning a week out when the temperature had been revised up. Had additional staff in, additional water points, additional measures to open the doors earlier, the usual stuff that (xxx) has put out.”* (Medical, 5).

### Legislation is a key component of event safety

Legislation is fundamental in event safety as it provides a structured and enforceable framework that ensures comprehensive risk management, legal accountability, public protection, contributing to the safe and successful execution of events.

*“We agree that there should be some form of legislation in place which provides the board mechanisms of reporting standards at events and some authority for review process to determine as to whether this event should occur for a number of reasons, public safety, and public management. If we have 20 events on one weekend then the standard community services that would be available to the community are going to be dramatically reduced hence because they are all tied up with these events”* (Police, 3).

*“…we went through all of our policies and compared them to the Act and regs and updated them, knowing that they would be adopted.”* (Event Manager, 2).

*“You should be looking at doing it by the regulations. Here’s the regulation’ or whatever it may be and providing that level of support. Then the regulators will come through when there’s a serious breach, which to me makes a lot more sense.”* (Event Manager, 1).

As well as legislation, desk top exercises and continuous quality improvement are a must to ensure event safety.

*“We are required to have a debrief and with that comes the opportunity for the good, the bad, the ugly to actually be identified and addressed, so recorded and addressed, and we use those as a constant quality assurance and performance management as well as something to review and make sure that were not making the same mistake or we are using a strategy, will it actually work really well for the next event?”* (Police, 2).

*Desktop exercise every year and rotate it between Royal Show specific and a non-Royal show event and take it to the extreme. Someone of the scenarios may be ridiculous but it highlights a broad range of policies every time we do it...//. it’s a good process to highlight deficiencies and what the policies are and where you can find them.”* (Event Manager, 3).

## Discussion

Participants in this study identified many risks to event safety, lack of risk assessment, communication, lack of ownership of risk and poor planning. Even though these risks were similar each participant group identified their own perspective with their own ways of managing them. All event stakeholders should focuses on the interests of both the audience and the broader event community, with the goal of working together in an effort to foster a safe, supportive and trusting environment for all (24–26). Similar to the harm minimisation space; strategies that are set in isolation are often less effective than safety strategies and policies that are used within a combined approach. This combined effort not only fosters collaboration in assessing risks, but also fosters the development of frameworks to reduce harm (18). This approach encourages all parties to have a voice, representing the motivations of all groups, and if agreed upon can increase the safety of the event, inside and out (27).

Current event safety policies generally focus on curtailing the activities of individuals and do little to reduce the burden of harm on the broader community. These types of policies can include those that relate to the health and safety of the event goers and workers at the event. The creation and adaptability of a single multi-organisational response that includes police, event managers and medical providers would enable better communication and coordination between all parties as well as sending one clear message to event goers (27).

An important outcome of a single policy is the opportunity for consistency in the incident and health-related documentation at an event leading to the collection of more evidence-based data that further informs and supports the management and development of strategies supporting event safety. Consistency, and the increased amount of data; can assist in the evaluation of event safety approaches at events and provide a more reliable analysis of the effectiveness of safety policies and practises on the health and safety of audience members and workers at events (27). This approach also assists in maintaining good collaborative practise amongst all participating organisations (18).

The event environment is a specific space where many risks and safety strategies can be applied to increase the safety of the event. Having an event safety strategy were all parties are invested in the safety of the event can lead to a preventative model of event management rather than a reactive environment. Prevention is socially and economically cheaper, and event organisers need to be prepared to invest the time necessary to develop and strengthen preventative strategies (28).

### Commentary: crowd management through three-way conversation

The case project highlighted addresses a specific need (identified in discussions with event managers, event medical professionals and police personnel) for an established and rigorous approach that responds in real time to changes in the broader event and which has strategies and techniques that can be applied to minimise and modify those behaviours to reduce risk and harm. Designing a safe event requires a more complete understanding of event management and audience behaviour. This understanding enables the event organiser to monitor a broad range of factors at the mass gathering event in real time. Then, when any factor or combination of factors reaches a critical point, the event manager acts to pre-emptively influence and modify audience behaviour before negative and/or unsafe behaviours are initiated. In a time of social fragility protocols such as those which allow for real-time flexibility are determined by trust and shared knowledge, wherein there is capacity (knowledge) in each stakeholder to “predict social actors’ behaviours across different contexts and situations” [(25), p. 1000].

**Figure 1:**
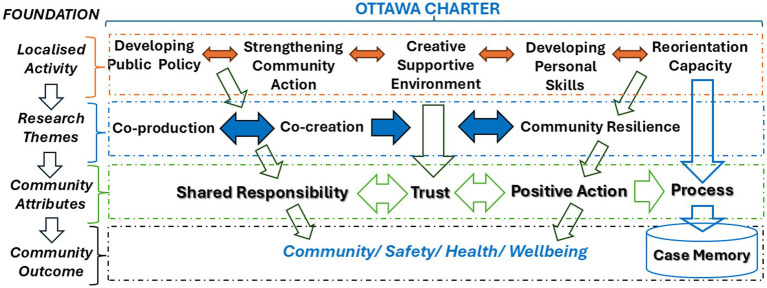
Framework for developing personal skill and community resilience for mass gathering events.

Building trust and shared knowledge requires a civic sense of commitment, factors that Putnam (29) forwarded as facilitating a better coordinated and well-functioning society (30). These are also elements of community that Bauman (31) sees as fleeting and evaporative in normal tourist activity (30). In tandem with the context of MGE, more is required to avoid distrust or the evaporation or erosion of trust (32) between those with responsibility for the safety of MGE crowds and the broader community, and to assure that outcomes are predicated on positive psychological benefits (33, 34) as well as physical safety. Building trust in the context of mass gathering events brings forward an opportunity for new orientation strategies which have been determined by a process much like that outline in Figure 1.

## Conclusion

Current event services are set up with a reliance on the existing community resources without taking into account how events can be used to promote harm minimisation health promotion and promote civic responsibility to lessen the need for supportive services thus reducing the burden on the broader community. The case study found that all services interviewed focused on event safety, but from their own siloed perspective, they did not understand the perspective or needs of other services.

Mass gathering events and smaller events are viewed in respect of their possible symbiosis with community. Additionally, events and other cultural and community activity can aid the capacity of citizens to adapt and contribute to positive social development and become more resilient. Reflecting on the critical juncture of society, set currently within a period of social and economic turbulence, accelerated by COVID-19 events should take on the board the task of civic responsibility. One of the more immediate pathways towards that civic duty is through a more dynamic and sympathetic process of crowd management as identified in the research captured here.

## Data Availability

The raw data supporting the conclusions of this article will be made available by the authors, without undue reservation.
